# Comparative effects of *Saccharomyces cerevisiae*, mannan-oligosaccharides, and their synbiotic on growth performance, gut morphology, immune responses, and economic efficiency in broiler chickens

**DOI:** 10.1016/j.psj.2026.106447

**Published:** 2026-01-13

**Authors:** Z. Li, F. Raziq, M. Waqas, S. Khan, T.A. Asseri, M. Mobashar, A. Ullah, H.A. Bachaya, M.T. Khan, N. Baazaoui, M. Al-Rasheed, S. Yang

**Affiliations:** a Qujing Normal University, College of Biological Resource and Food Engineering, 655011 Yunnan, China; b Department of Poultry Production, Faculty of Animal Production and Technology, University of Veterinary and Animal Sciences, Lahore 54000, Pakistan; c Department of Poultry Science, Faculty of Animal Husbandry and Veterinary Sciences, The University of Agriculture, Peshawar 25130, Pakistan; d Department of Poultry Science, Faculty of Animal Production and Technology, Cholistan University of Veterinary and Animal Sciences, Bahawalpur 63100, Pakistan; e Central Diagnostic Laboratory, Cholistan University of Veterinary and Animal Sciences, Bahawalpur 63100, Pakistan; f Department of Animal Nutrition, Faculty of Animal Husbandry and Veterinary Sciences, The University of Agriculture, Peshawar 25130, Pakistan; g Institute of Microbiology, Faculty of Veterinary Sciences, University of Veterinary and Animal Sciences, Lahore 54000, Pakistan; h Livestock and Dairy Development Department, Poultry Research Institute, Rawalpindi 46300, Pakistan; i Department of Biology, College of Science, King Khalid University, Abha 61413, Saudi Arabia; j Central Labs, King Khalid University, AlQura'a, Abha, P.O. Box 960, Saudi Arabia; k Biology Department, Faculty of Science, King Khalid University, Abha, Saudi Arabia; l Tissue Culture and Cancer Biology Research Laboratory, King Khalid University, Abha 9004, Saudi Arabia; m Department of Clinical Sciences, College of Veterinary Medicine, King Faisal University, 31982 Al-Ahsa, Saudi Arabia

**Keywords:** *Saccharomyces cerevisiae*, Synbiotic, Growth performance, Gut health, Immune response

## Abstract

In recent years, the use of *Saccharomyces cerevisiae* (SC) as a probiotic, its cell-wall derived mannan-oligosaccharides (MOS) as a prebiotic, and their combined application as a synbiotic has gained recognition as an effective nutritional strategy to replace the non- therapeutic use of antibiotics in animal feed. The present study investigated the effects of dietary supplementation with SC, MOS, and their combination (SC+MOS) on growth performance, intestinal morphology, hematological indices, immune responses, and economic efficiency in Ross-308 broiler chickens. A total of 200 day-old chicks were randomly assigned to four dietary treatments in a completely randomized design, with five replicates of ten birds each. The dietary treatments included a basal diet (T₁, control), SC at 1 g/kg feed (T₂), MOS at 1 g/kg feed (T₃), and a synbiotic combination of SC and MOS at 0.5 g/kg each (T₄). Birds receiving the synbiotic diet exhibited significantly greater cumulative feed intake and body weight gain, along with the most efficient feed conversion ratio, compared to the control and other supplemented groups (*P <* 0.001). Intestinal morphology was markedly improved in synbiotic-fed birds (*P <* 0.001), as evidenced by increased villus height, reduced crypt depth, and a higher villus height-to-crypt depth ratio. Hematological parameters, including red blood cell count, hemoglobin concentration, and packed cell volume, were significantly elevated in the probiotic group (*P <* 0.001), whereas white blood cell counts were highest in the prebiotic group. Both humoral and innate immune responses were enhanced, as indicated by higher Newcastle disease antibody titers in the synbiotic and probiotic groups (*P <* 0.01) and increased phagocytic activity in synbiotic-fed birds (*P <* 0.001). Economically, synbiotic supplementation resulted in the greatest profitability, followed by prebiotic, probiotic, and control diets. Taken together, these findings demonstrate that synbiotic supplementation confers superior improvements in growth performance, intestinal health, immune competence, and economic returns, supporting its use as a sustainable and effective feed additive in modern broiler production systems.

## Introduction

The rapid expansion and intensification of poultry production systems have increased the demand for nutritional strategies that simultaneously enhance growth performance, preserve intestinal integrity, and support immune competence, particularly in the context of reduced or banned use of antibiotic growth promoters. Growing concerns over antimicrobial resistance, food safety, and consumer acceptance have accelerated the global shift toward natural, biologically active feed additives—including probiotics, prebiotics, and synbiotics—as sustainable alternatives for broiler nutrition (Khalid et al., 2021; Okey et al., 2023; Salahi and Abd El‐Ghany, 2024; Soren et al., 2024; Susalam et al., 2024; Abd El-Ghany, 2025). The adoption of these alternatives has also alleviated consumer concerns regarding antibiotic residues and the spread of antimicrobial resistance through poultry-derived products (Hoque et al., 2021).

Probiotics are defined as live microorganisms that, when administered in adequate amounts, confer health benefits to the host by modulating gastrointestinal microbiota, enhancing gut barrier function, and regulating immune responses (Hutkins et al., 2016; Reid, 2016; Hu, 2024). Among probiotic candidates, *Saccharomyces cerevisiae* (**SC**), a non-pathogenic yeast, has garnered significant attention due to its thermal stability during feed processing, resistance to gastrointestinal stress, and multifunctional biological activities (Patterson et al., 2023). Dietary supplementation with SC has been shown to stimulate digestive enzyme activity, enhance nutrient digestibility, and improve intestinal morphology by increasing villus height and the villus-to-crypt depth ratio, thereby promoting more efficient nutrient absorption and improved feed conversion efficiency in broiler chickens (Patane et al., 2017; Kim et al., 2022; Comi et al., 2025). Additionally, SC can suppress enteric pathogens through competitive exclusion, pathogen agglutination, and the production of antimicrobial metabolites, collectively contributing to enhanced gut health and performance in antibiotic-free production systems (Lin et al., 2023; Raza et al., 2024; Srifani et al., 2024; Mehmood et al., 2025; Naeem and Bourassa, 2025).

Prebiotics, in contrast, are non-digestible dietary components that selectively stimulate the growth and metabolic activity of beneficial intestinal microorganisms (Bozkurt et al., 2014; Dev et al., 2020). Mannan-oligosaccharides (**MOS**), derived from the cell wall of SC, are among the most extensively studied prebiotics in poultry nutrition (Ferket, 2003). The primary mode of action of MOS involves binding to type-1 fimbriae of pathogenic bacteria, such as *Escherichia coli* and *Salmonella* spp., which prevents their adhesion to the intestinal epithelium and facilitates their removal from the gastrointestinal tract (Fadl et al., 2020). Moreover, MOS supplementation has been shown to enhance intestinal morphology, promote a balanced gut microbiota, and reduce intestinal inflammation, collectively contributing to improved growth performance and feed efficiency in broiler chickens (Patterson et al., 2023; Berberoglu et al., 2025; Nastoh et al., 2025; Polidoro et al., 2025).

The synbiotic concept, which combines probiotics and prebiotics in a single formulation, has emerged as a promising nutritional strategy to maximize the functional benefits of both components (Dev et al., 2020). By providing a selective substrate for probiotic microorganisms, synbiotics enhance their survival, colonization, and metabolic activity within the gastrointestinal tract, leading to synergistic effects on gut microbiota composition, intestinal development, and immune modulation (Tuohy et al., 2003). Recent studies have demonstrated that synbiotic supplementation often results in greater improvements in body weight gain, feed conversion ratio, intestinal histomorphology, and immune responsiveness compared to probiotic or prebiotic supplementation alone (Soren et al., 2024; Khosravi et al., 2025). These synergistic effects are particularly relevant in modern broiler production systems, where optimal intestinal health and robust immune resilience are critical determinants of productivity.

Beyond their localized effects on the gastrointestinal tract, dietary supplementation with SC, MOS, and synbiotics has been shown to positively influence hematological parameters and systemic immune responses. By enhancing gut integrity and reducing pathogen burden, these feed additives can alleviate metabolic stress and inflammatory load, leading to more stable hematological profiles and improved humoral and cell-mediated immunity (Wang et al., 2021). Additionally, broilers receiving yeast-based feed additives have demonstrated increased antibody titers against common poultry diseases and favorable modulation of cytokine expression, further highlighting the immunomodulatory potential of these supplements (Hoque et al., 2021).

Despite extensive evidence supporting the individual benefits of probiotics and prebiotics, direct comparative evaluations of SC, MOS, and their synbiotic combination under identical experimental conditions are limited, particularly in the challenging environmental conditions prevalent in Pakistan. Therefore, the present study was designed to systematically assess the effects of dietary supplementation with SC as a probiotic, MOS as a prebiotic, and their combination as a synbiotic on growth performance, intestinal health, hematology, and immune responses in broiler chickens.

## Materials and methods

### Location and duration of experiment

The study was conducted at the Poultry Research Unit, Department of Poultry Science, Faculty of Animal Husbandry and Veterinary Sciences, University of Agriculture, Peshawar (**UAP**), Pakistan (coordinates: 34.0151°N, 71.5249°E; altitude: 331 m). The region has a tropical climate, characterized by hot and humid conditions, with ambient temperatures ranging from approximately 5 °C in winter to 45 °C in summer. All experimental procedures were conducted in accordance with ethical standards approved by the UAP Institutional Ethics Committee (Approval No. 4306/LM & ABG/UAP; 31 May 2017) and adhered to national and institutional guidelines for the care and use of animals. The feeding trial lasted 42 days, which included a one-week adaptation period for the chicks prior to the start of the experiment.

### Experimental birds and Husbandry

A total of 200 day-old Ross-308 broiler chicks, with a uniform initial body weight of 43.50 ± 0.50 g, were randomly assigned to four dietary treatments (T₁–T₄) in a completely randomized design (**CRD**), comprising five replicates of ten birds each (Table 1). The dietary treatments included a basal diet (T₁, control), SC at 1 g/kg feed (T₂), MOS at 1 g/kg feed (T₃), and a synbiotic combination of SC and MOS at 0.5 g/kg each (T₄). The trial was conducted in an open-sided poultry house (30 ft × 40 ft × 10 ft), where the birds were housed in 20 wire-mesh floor pens (3 ft × 3 ft × 3 ft) bedded with fresh, dry wood shavings to a depth of 3.5 cm. Prior to chick placement, all pens, floors, and equipment were thoroughly cleaned and disinfected, and the entire facility was fumigated with formaldehyde five days before the chicks’ arrival. Brooding temperature was maintained at 32 ± 3 °C during the first week and was then reduced by 2 °C each week until reaching 26°C by the fourth week, which was maintained for the remainder of the trial. Relative humidity was kept at 65 ± 5 %. Continuous lighting was provided for the first 24 h, followed by a schedule of 23 h of light and 1 h of darkness until the end of the experiment. Birds were fed iso-caloric and iso-nitrogenous corn-soybean meal-based basal diets formulated to meet the nutritional requirements of Ross-308 broilers (Aviagen, 2016) and offered in mash form (Table 2). Feed and water were provided ad libitum throughout the 42-day experimental period. Each pen was equipped with two feeders and one nipple drinker, with their heights adjusted according to the birds’ age and growth stage.

**Table 1:** Experimental design.

Group	Treatment^1^	No. of birds
T_1_	Basal diet without supplements (control)	50
T_2_	Basal diet with SC at 1g/k of feed	50
T_3_	Basal diet with MOS at 1g/k of feed	50
T_4_	Basal diet with SC + MOS each at 0.5g/kg of feed	50

1 SC: Saccharomyces cerevisiae, MOS: mannan-oligosaccharides.

**Table 2:** Composition of the basal diet for starter and finisher phases.

Ingredients (%)	Starter phase (1–21 days)	Finisher phase (22–42 days)
Maize	53.50	58.19
Soybean meal (44 % CP)	36.00	31.00
Fish meal (55 % CP)	3.00	2.00
Vegetable oil	3.50	5.00
Dicalcium phosphate	1.80	1.75
Limestone	1.10	1.00
Sodium chloride	0.30	0.30
DL-Methionine	0.13	0.12
Threonine	0.07	0.04
Sodium bicarbonate	0.10	0.10
Vitamin premix^1^	0.20	0.20
Minerals premix^2^	0.30	0.30
Total	100	100
Nutrient		
Metabolizable energy (kcal/kg)	3000	3150
Crude protein	22.00	20.00
Calcium	0.90	0.88
Available phosphorus	0.45	0.40
Lysine	1.20	1.10
Methionine	0.50	0.45

1 Provided per kg of diet: vitamin A, 11,000 IU; vitamin D_3_, 2,560 IU; vitamin E, 44 IU; vitamin K, 4.2 mg; riboflavin, 8.5 mg; niacin, 48.5 mg; thiamine, 3.5 mg; D-pantothenic, 27 mg; choline, 150 mg; vitamin B_12_, 33 μg.

2 Provided per kg of diet: manganese, 60 mg; zinc, 70 mg; iron, 30 mg; copper, 10 mg; iodine, 0.45 mg; selenium, 0.2 mg.

### Feed supplements

The probiotic SC preparation was produced under controlled fermentation and drying conditions at the Centre of Biotechnology and Microbiology, UAP, Pakistan, following standardized microbial culture protocols that have been previously validated for high cell viability, stability, and reproducibility (Markowiak and Śliżewska, 2018). The prebiotic MOS, derived from the yeast cell wall, was sourced from a certified commercial supplier (Bio-Mos®, Alltech Inc., Nicholasville, KY, USA). For synbiotic supplementation, SC and MOS were blended in equal weight proportions to ensure uniform distribution of viable yeast cells and prebiotic substrates, thereby maximizing their potential synergistic effects (Rehman et al., 2020). All experimental diets were prepared in mash form without pelleting or thermal processing to prevent heat-induced loss of probiotic viability. The SC product used was a live yeast probiotic with a viable count of ≥1 × 10⁹ CFU/g at the time of dietary inclusion. Throughout the experimental period, the probiotic was stored in airtight containers under cool, dry conditions to maintain microbial stability and viability.

### Data collection

#### Growth performance

Chicks were individually weighed at placement and subsequently at weekly intervals using a digital balance. Body weight gain (**BWG**) was calculated as the difference between the final body weight at day 42 and the initial body weight. All body weight measurements were taken in the morning prior to feed access to minimize variability associated with gut fill. Feed intake (**FI**) was determined on a pen basis by subtracting the residual feed from the total feed offered during each measurement period. The feed conversion ratio (**FCR**) was calculated on a pen basis as the ratio of cumulative feed intake (**CFI**) to total BWG. Mortality was recorded daily and used to adjust FI and FCR calculations accordingly. The mortality rate was expressed as the percentage of birds that died relative to the initial number of chicks in each treatment group.

#### Gut morphology

At the end of the experimental period, four birds from each replicate were randomly selected and humanely slaughtered using the Halal method (Farouk et al., 2014). Approximately 1 cm-long segments of the jejunum were collected from each bird and immediately fixed overnight in 10 % neutral-buffered formalin. The fixed tissues were then processed and sent to a histopathology laboratory for routine slide preparation. For each jejunal sample, three well-oriented villi were selected for microscopic evaluation. Villus height (**VH**), crypt depth (**CD**), and the villus height-to-crypt depth ratio (**VH:CD**) were measured for each villus following the method described by Panda et al. (2009).

#### Hematological profile and immune response

During slaughter, blood samples (3 mL) were collected from four birds per replicate into EDTA-containing tubes for hematological analysis. Hematological parameters, including red blood cell (**RBC**) count, white blood cell (**WBC**) count, packed cell volume (**PCV**), and hemoglobin (**Hb**) concentration, were measured using an automated hematology analyzer (Cell-DYN 3500; Abbott Diagnostics, USA). For immunological assessments, an additional set of blood samples was collected into plain tubes without anticoagulant and centrifuged at 4,000 × *g* for 15 min at 4°C to obtain serum, which was then stored at −20°C until analysis. Antibody titers against Newcastle disease (**ND**) virus were assessed using the hemagglutination inhibition (**HI**) assay with commercial diagnostic kits (BioChek, Gouda, the Netherlands). The phagocytic activity of macrophages was evaluated using the carbon clearance assay as described by Ghally and El-Latif (2010). All birds had been vaccinated against ND with live attenuated vaccines according to the manufacturer’s instructions, with vaccination performed one week prior to blood collection.

#### Economic evaluation

At the conclusion of the trial, a comprehensive economic analysis was conducted to evaluate the impact of dietary supplementation with SC as a probiotic, prebiotic, or synbiotic. This analysis considered factors such as feed intake, additive cost and inclusion level, mortality, final body weight, and prevailing market prices. Total production costs were calculated by summing feed, additive, and fixed rearing expenses, while revenue was determined by multiplying the final live body weight by the market price. Following the methodologies of van Wagenberg et al. (2020) and Kamruzzaman et al. (2021), net profit and cost per kilogram of weight gain were also calculated, providing an integrated measure of the efficiency and economic viability of each dietary treatment.

#### Statistical analysis

Data were analyzed using a one-way analysis of variance under CRD, using SAS software (SAS Institute Inc., Cary, NC, 2002–2003). Differences among treatment means were assessed using Duncan’s Multiple Range Test, with statistical significance set at *P <* 0.05. Each pen was treated as the experimental unit. The statistical model employed was:

Yij=μ+τi+εij

Where: Yij = Observed dependent variable; μ = Overall mean; τi = Effect of the ith treatment; and εij = Residual error.

## Results

### Growth performance

The effects of dietary supplementation with SC, MOS, and their combination (SC+MOS) on broiler growth performance are presented in Table 3. Dietary treatments significantly affected all growth performance parameters except mortality, which remained unaffected. Birds receiving the synbiotic diet exhibited the highest CFI and BWG, followed by those fed the prebiotic and probiotic diets, whereas the control group recorded the lowest values. FCR was also significantly improved (P < 0.001) by biotic supplementation, with the synbiotic group achieving the most efficient FCR, followed by the prebiotic and probiotic treatments, while the control birds showed the poorest feed efficiency.

**Table 3:** Effects of *Saccharomyces cerevisiae* supplementation in different biotic forms on the growth performance of broiler chickens at 42 days of.age.

Treatments^2^	Parameters^1^			
	CFI (g)	WG (g)	FCR	M (%)
T_1_	3306^d^	1851^d^	1.79^a^	0.3
T_2_	3423^c^	2007^c^	1.71^b^	0.3
T_3_	3517^b^	2069^b^	1.70^b^	0.0
T_4_	3665^a^	2225^a^	1.65^c^	0.0
SEM	6.201	4.382	0.028	0.001
P-value	0.0001	0.0001	0.0001	0.59

^a-d^Different superscripts on means within a column show significant difference (*P <* 0.05).

1 CFI: cumulative feed intake, WG: weight gain, FCR: feed conversion ratio, M: mortality.

2 T_1_: basal diet (control); T_2_: basal diet with SC at 1g/kg of feed; T_3_: basal diet with MOS at 1g/kg of feed; T_4_: basal diet with SC + MOS each at 0.5g/kg of feed.

### Gut morphology

Table 4 illustrates the effects of dietary supplementation with SC, MOS, and their combination (SC+MOS) on broiler intestinal morphology. The dietary treatments significantly affected gut structure. Birds fed the synbiotic diet exhibited the greatest VH (*P <* 0.001), followed by those receiving the prebiotic and probiotic diets, while the control group had the lowest values. In contrast, the CD was shallowest (*P <* 0.001) in the synbiotic group, followed by the prebiotic, probiotic, and control treatments. As a result, the VH:CD ratio was highest in the synbiotic group (*P <* 0.001), followed by the prebiotic and probiotic groups, with the control birds showing the lowest ratio.

**Table 4:** Effects of *Saccharomyces cerevisiae* supplementation in different biotic forms on histomorphology of jejunum in broiler chickens.

Treatments^2^	Parameters^1^		
	VH (µm)	CD (µm)	VH:CD
T_1_	1530^d^	230^a^	6.65^d^
T_2_	1565^c^	218^b^	7.18^c^
T_3_	1590^b^	198^c^	8.03^b^
T_4_	1620^a^	186^d^	8.71^a^
SEM	9.062	7.512	0.032
P-value	0.0001	0.0001	0.0001

^a-d^Different superscripts on means within a column show significant difference (*P <* 0.05).

1 VH: villus height, CD: crypt depth.

2 T_1_: basal diet (control); T_2_: basal diet with SC at 1g/kg of feed; T_3_: basal diet with MOS at 1g/kg of feed; T_4_: basal diet with SC + MOS each at 0.5g/kg of feed.

### Hematological profile

The effects of dietary supplementation with SC, MOS, and their combination (SC+MOS) on broiler blood parameters are presented in Table 5. Dietary treatments significantly impacted all hematological indices. Birds receiving the probiotic diet exhibited the highest RBC counts, Hb concentration, and PCV compared to the control and other dietary groups (*P <* 0.001). In contrast, WBC counts were significantly highest (*P <* 0.001) in the prebiotic group, followed by the synbiotic, probiotic, and control treatments.

**Table 5:** Effects of *Saccharomyces cerevisiae* supplementation in different biotic forms on hematological parameters and immune response of broiler chickens at 42 days of age.

Treatments^2^	Hematology^1^			Antibody titer^1^		Phagocytic assay (%)	
	RBC × 10^6^	WBC × 10^3^	Hb (g/dL)	PCV (%)	ND (HI titer, log_2_)	At 3 Minutes	At 15 Minutes
T_1_	1.90^c^	136^d^	9.50^c^	23.6^c^	3.30^b^	69.6^d^	84.70^d^
T_2_	2.40^a^	155^c^	11.90^a^	28.9^a^	5.70^a^	73.70^b^	88.50^b^
T_3_	2.30^b^	159^a^	11.40^b^	27.4^b^	4.30^b^	71.90^c^	85.80^c^
T_4_	2.30^b^	158^b^	11.70^ab^	27.1^b^	6.30^a^	78.60^a^	92.40^a^
SEM	0.002	2.628	0.844	0.721	0.002	1.016	1.042
P-value	0.0001	0.0001	0.0001	0.0001	0.0009	0.0001	0.0001

^a-d^Different superscripts on means within a column show significant difference (*P <* 0.05).

1 RBC: red blood cells, WBC: white blood cells, Hb: hemoglobin, PCV: packed cell volume, ND: Newcastle disease.

2 T_1_: basal diet (control); T_2_: basal diet with SC at 1g/kg of feed; T_3_: basal diet with MOS at 1g/kg of feed; T_4_: basal diet with SC + MOS each at 0.5g/kg of feed.

### Immune response and phagocytic activity

The effects of dietary supplementation with SC, MOS, and their combination (SC+MOS) on the immune response and phagocytic activity of broilers are presented in Table 5. Dietary treatments significantly influenced both humoral and innate immunity. Newcastle disease antibody titers were significantly highest in the synbiotic and probiotic groups compared to the control and prebiotic groups (*P <* 0.01). Similarly, phagocytic activity measured at 3 and 15 min was greatest in the synbiotic group, followed by the probiotic, prebiotic, and control groups (*P <* 0.001).

### Broiler production economics

Table 6 illustrates the effects of dietary supplementation with SC, MOS, and their combination (SC+MOS) on the economics of broiler production. All supplemented groups significantly improved production profitability. Although feed and additive costs were slightly higher in the supplemented groups, these costs were offset by increased gross returns and net profits. The synbiotic group achieved the highest gross return and net profit, followed by the prebiotic and probiotic groups, while the control group recorded the lowest values.

**Table 6:** Effect of *Saccharomyces cerevisiae* supplementation in different biotic forms on broiler production economics.

Treatments^3^	Parameters^1^^,^^2^					
	FC	SC	OC	TE	GR	NP
T_1_	149^d^	0.00^d^	50	199^d^	307^d^	108^d^
T_2_	154^c^	2.70^a^	50	207^c^	333^c^	127^c^
T_3_	158^b^	2.20^c^	50	211^b^	343^b^	133^b^
T_4_	165^a^	2.60^b^	50	218^a^	369^a^	151^a^
SEM	2.002	0.001	0.000	3.108	3.248	2.018
P-value	0.0001	0.0001	0.0001	0.0001	0.0001	0.0001

^a-d^Different superscripts on means within a column show significant difference (*P <* 0.05).

1 FC: feed cost, SC: supplement cost, OC: operational cost, TE: total expenditure, GR: gross return, NP: net profit.

2 Every value is displayed in PKR (Pakistani rupee).

3 T_1_: basal diet (control); T_2_: basal diet with SC at 1g/kg of feed; T_3_: basal diet with MOS at 1g/kg of feed; T_4_: basal diet with SC + MOS each at 0.5g/kg of feed.

## Discussion

### Growth performance

The present findings clearly demonstrate that the combined supplementation of SC and MOS (synbiotic) significantly enhances broiler growth performance without adversely affecting survivability. The synbiotic group exhibited the highest CFI and BWG, along with the most efficient FCR, indicating improved nutrient utilization efficiency in supplemented birds. Mechanistically, these improvements can be attributed to SC’s ability to positively modulate the intestinal microbiota by promoting beneficial microbial populations and suppressing enteric pathogens, thereby enhancing gut health and digestive efficiency (Patane et al., 2017; Comi et al., 2025). Additionally, MOS derived from the yeast cell wall serve as competitive binding sites for pathogenic bacteria, reducing their adhesion to the intestinal epithelium and facilitating their removal from the gastrointestinal tract (Sun et al., 2020; Khalid et al., 2021). The superior performance observed in birds fed synbiotics can be attributed to the synergistic interactions between live yeast and MOS. These interactions beneficially modulate the intestinal microbiome and enhance the production of short-chain fatty acids, providing additional energy to enterocytes (Bortoluzzi et al., 2018). As a result, metabolic nutrient losses are minimized, and overall nutrient utilization efficiency is improved, leading to a more effective conversion of feed into body tissue, as evidenced by the improved FCR (Dev et al., 2020; Khalid et al., 2021; Rauf et al., 2024). Importantly, the absence of significant differences in mortality among dietary treatments indicates that these performance improvements were achieved without compromising physiological stability or bird welfare, thus confirming the safety of the supplemented diets.

These findings are consistent with previous studies demonstrating that supplementation with SC, MOS, or synbiotic formulations improves growth performance, feed efficiency, intestinal morphology, and the composition of beneficial cecal microbiota in broiler chickens (Li et al., 2016; Gao et al., 2017; Wang et al., 2017; Ding et al., 2019; Dev et al., 2020; Comi et al., 2025). Hoque et al. (2021) and Attia et al. (2023) likewise reported that including SC in broiler diets increased BWG and improved FCR compared to control diets. In line with these observations, Hossain et al. (2024) demonstrated that BWG, FI, and FCR were significantly affected (*P <* 0.05) by MOS and yeast supplementation across all treated groups compared to antibiotic-treated and unsupplemented controls. Notably, accumulating evidence indicates that synbiotic diets produce more pronounced improvements in growth performance and gut health than either probiotic or prebiotic supplementation alone, underscoring the additive and synergistic benefits of their combined use (Rauf et al., 2024; Soren et al., 2024; Khosravi et al., 2025). Additionally, yeast-MOS combinations have been shown to reduce intestinal pathogenic bacterial loads and stabilize gut microbial ecology, thereby promoting improved nutrient utilization and overall production efficiency (Khalid et al., 2021; Rauf et al., 2024). While responses may vary depending on yeast strain, inclusion level, and dietary context, the existing body of evidence strongly supports yeast-based biotics as effective, safe, and antibiotic-free feed additives for improving broiler productivity (Attia et al., 2023; Soren et al., 2024; Comi et al., 2025; Khosravi et al., 2025).

### Gut morphology

The present study demonstrates that the combined supplementation of SC and MOS as a synbiotic significantly enhances intestinal morphology in broiler chickens. Birds fed the synbiotic diet exhibited the greatest VH, the shallowest CD, and the highest VH:CD ratio compared to the control and other dietary treatments. These favorable architectural changes indicate reduced epithelial cell turnover and renewal, thereby lowering the metabolic energy required for intestinal tissue maintenance. Increased VH and the VH:CD ratio, along with reduced CD, are widely recognized as indicators of a more mature and functionally efficient intestinal mucosa, in which cellular activity is preferentially directed toward nutrient absorption rather than excessive proliferation. Consequently, the VH:CD ratio serves as a robust indicator of intestinal development and functional capacity, with higher values reflecting an expanded absorptive surface area for nutrient digestion and uptake (Gao et al., 2008). Improvements in intestinal structure are closely associated with enhanced feed utilization, growth performance, and overall health in broilers (Santin et al., 2001; Sugiharto, 2016; Fu et al., 2019). Mechanistically, SC promotes a favorable gut microbial balance by stimulating beneficial bacterial populations while suppressing enteric pathogens, thereby alleviating intestinal inflammation and supporting mucosal integrity and development (Haldar et al., 2011; Javadi et al., 2012; Comi et al., 2025). Concurrently, MOS act as competitive binding sites for pathogenic bacteria, preventing their adhesion to the intestinal epithelium and reducing crypt hyperplasia, which plausibly explains the shallower crypts observed in supplemented birds (Spring et al., 2000; Sun et al., 2020; Khalid et al., 2021; Kyoung et al., 2023).

The superior intestinal responses observed in synbiotic-fed birds likely result from synergistic interactions between live yeast and MOS, which enhance microbial fermentation and short-chain fatty acid production—key energy substrates for enterocytes—thereby promoting villus elongation and epithelial differentiation (Dev et al., 2020; Fathima et al., 2022; Rauf et al., 2024; Khosravi et al., 2025). These morphological adaptations collectively reduce metabolic energy losses associated with excessive crypt cell turnover while increasing the absorptive surface area, leading to more efficient nutrient utilization and improved growth performance. Consistent with these findings, similar enhancements in VH, VH:CD ratio, and overall gut health have been reported in broilers fed yeast-based synbiotics compared to those receiving only probiotic or prebiotic supplementation (Awad et al., 2009; Khalid et al., 2021; Khogali et al., 2022; Assaf et al., 2025; Khosravi et al., 2025). By improving intestinal structure and function, synbiotic supplementation enhances nutrient absorption, stabilizes gut microbial ecology, and limits pathogen colonization, thereby supporting superior growth performance and feed efficiency in broiler chickens (Comi et al., 2025; Khosravi et al., 2025).

### Hematological profile

Combined supplementation of SC and MOS as a synbiotic significantly improved hematological parameters in broiler chickens, including RBC counts, Hb concentration, PCV, and WBC counts. Mechanistically, SC improves erythropoiesis and hemoglobin synthesis by enhancing the intestinal absorption and bioavailability of key nutrients, such as iron, B-complex vitamins, and essential amino acids, which are critical for red blood cell production and efficient oxygen transport. Concurrently, MOS acts as a prebiotic by selectively promoting beneficial gut microbiota while suppressing enteric pathogens, thereby reducing intestinal inflammation and stimulating immune activation, as evidenced by elevated WBC counts (Khalid et al., 2021; Sun et al., 2020; Kyoung et al., 2023; Comi et al., 2025). The superior hematological responses observed in synbiotic-fed birds likely result from the synergistic interactions between live yeast and MOS, which simultaneously enhance nutrient utilization and modulate the gut microbial ecosystem, supporting both erythropoiesis and innate immune function. Additionally, microbial metabolites, such as short-chain fatty acids produced during fermentation, serve as energy substrates for enterocytes and immune cells, further supporting blood cell proliferation and immune competence (Dev et al., 2020; Kyoung et al., 2023; Rauf et al., 2024; Assaf et al., 2025; Khosravi et al., 2025). Collectively, these coordinated effects optimize oxygen transport, enhance pathogen defense, and improve overall physiological resilience in supplemented birds.

These findings are consistent with recent studies demonstrating that probiotic and synbiotic supplementation significantly improves RBC, Hb, PCV, and WBC profiles in broilers, thereby supporting enhanced growth performance, immune status, and general health (Khalid et al., 2021; Soren et al., 2024; Assaf et al., 2025; Khosravi et al., 2025; Comi et al., 2025). Likewise, Attia et al. (2022) reported that dietary SC supplementation increased PCV, Hb, RBC counts, and key leukocyte populations, including lymphocytes, monocytes, and heterophils. Furthermore, growing evidence indicates that synbiotic formulations consistently outperform individual probiotic or prebiotic applications, reflecting additive and synergistic effects that maximize nutrient utilization and immune functionality (Khosravi et al., 2025; Attia et al., 2023). Overall, these studies confirm that SC-based biotics, particularly when used as synbiotics, represent effective, safe, and antibiotic-free dietary strategies for enhancing hematological health and immune competence in modern broiler production systems.

### Immune response and phagocytic activity

Dietary supplementation with SC in probiotic, prebiotic, and synbiotic forms significantly enhanced both humoral and innate immune responses in broiler chickens. The elevated ND antibody titers observed in the synbiotic and probiotic groups indicate a strengthened adaptive immune response, likely mediated by improved antigen presentation, enhanced B-lymphocyte activation, and increased immunoglobulin synthesis. These responses can be attributed to the immunomodulatory components of yeast, including mannans, β-glucans, and nucleotides, which have consistently been shown to enhance serum antibody titers against viral diseases in poultry (Gao et al., 2008; Oliveira et al., 2009; Ghosh et al., 2012). In particular, yeast-derived β-glucans and MOS interact with pattern-recognition receptors such as dectin-1 and Toll-like receptors on macrophages and dendritic cells, thereby activating downstream cytokine signaling pathways that support both humoral and cell-mediated immunity (Wang et al., 2021; Teng et al., 2018; Rajput et al., 2014). Collectively, these immunostimulatory mechanisms enhance vaccine responsiveness and immune memory, ultimately improving resistance to viral challenges (Abd El-Ghany, 2025).

The significant increase in phagocytic activity at both early (3 min) and late (15 min) intervals across all supplemented groups—most pronounced in synbiotic-fed birds—further indicates enhanced innate immune competence and pathogen clearance capacity. This response is primarily linked to increased macrophage and heterophil activity induced by yeast polysaccharides and microbial metabolites (Chae et al., 2006; Rajapakse et al., 2010). Moreover, SC and MOS beneficially modulate the gut microbiota by selectively promoting beneficial bacterial populations while suppressing pathogenic organisms, thereby reducing intestinal endotoxin load and alleviating systemic immune stress. The enhanced microbial fermentation associated with synbiotic supplementation increases short-chain fatty acid production, which provides critical energy substrates for immune cells and plays a key role in promoting phagocytosis, regulating inflammatory responses, and maintaining mucosal immune integrity (Bi et al., 2022).

The superior immune responses observed in synbiotic-fed birds underscore the synergistic interaction between live SC and MOS, wherein the probiotic component directly stimulates immune activity, while the prebiotic selectively supports the growth and persistence of beneficial microbiota. This dual mechanism enhances communication along the gut–immune axis and amplifies immune signaling more effectively than either probiotic or prebiotic supplementation alone. Supporting this concept, Schwartz and Vetvicka (2021); Bi et al. (2022), and Cao et al. (2023) reported that yeast oligo-mannans and β-glucans enhance immune cell circulation, increase goblet cell density and mucin-2 production, upregulate intestinal tight-junction protein expression, and exert anti-inflammatory immunomodulatory effects in poultry. In line with the present findings, recent studies have demonstrated that synbiotic supplementation leads to greater improvements in ND antibody titers, phagocytic activity, and overall immune resilience compared to individual biotic applications (El-Manawey et al., 2021; Attia et al., 2022; Ogwiji et al., 2024). Likewise, Attia et al. (2022) reported an enhanced phagocytic index and ND-specific immune response in broilers supplemented with SC. In contrast, the inconsistent immune responses observed in some studies (e.g., Silva et al., 2009) may be attributed to variations in environmental stressors, yeast dosage, or experimental conditions, emphasizing the importance of optimal formulation and management strategies to fully realize the immunological benefits of yeast-based biotics.

### Broiler production economics

Dietary supplementation with SC in probiotic, prebiotic (MOS), and synbiotic forms significantly enhanced the production profitability of broiler chickens. Although feed and additive costs were slightly higher in the supplemented groups, these additional expenditures were effectively offset by increased gross returns and net profits. Notably, birds fed the synbiotic diet achieved the highest gross return and net profit among all dietary treatments, while the control group exhibited the poorest economic indices. The superior economic performance of synbiotic-fed birds can be attributed to improved production efficiency, driven by enhanced growth rates, superior feed conversion efficiency, and better overall health. By favorable modulating the intestinal microbiota and improving digestive functionality, SC enhances nutrient digestibility, metabolic efficiency, and body weight gain, thereby increasing marketable output per unit of feed consumed. Enhanced nutrient absorption and metabolic utilization reduce the cost per unit of live weight gain, directly contributing to improved profitability.

Probiotic yeast, such as SC, has been shown to stimulate digestive enzyme activity, stabilize intestinal microbial populations, and enhance nutrient uptake, collectively leading to improved growth performance and economic efficiency in commercial broiler production (Attia et al., 2023). Similarly, the prebiotic MOS contributes to better economic outcomes by reducing intestinal pathogenic load, preserving gut integrity, and minimizing production losses associated with subclinical enteric dysfunction, while also improving flock uniformity—an important determinant of consistent market weights and predictable economic returns (Asif et al., 2022). The synbiotic combination of SC and MOS further amplifies these benefits through synergistic interactions, wherein live SC enhances gut functionality and metabolic efficiency, while MOS selectively supports the establishment and persistence of beneficial microbial populations. This synergy results in greater nutrient assimilation, improved feed conversion ratios, reduced variability in growth performance, and ultimately higher returns on investment.

Consistent with the current findings, previous studies have shown that synbiotic supplementation yields superior economic outcomes compared to probiotic or prebiotic supplementation alone, primarily due to improved feed efficiency and reduced production risks (Saiyed et al., 2015; Kamel and Mohamed, 2016). Recent evidence further supports these observations; a meta-analysis of yeast-derived mannan-rich fractions demonstrated enhanced production efficiency and reductions in both feed and environmental costs through better nutrient utilization (Salami et al., 2024). Similarly, recent studies on SC fermentation products and MOS supplementation have reported improvements in growth performance, feed efficiency, carcass yield, and overall economic returns, reinforcing the role of yeast-based biotics as cost-effective and sustainable growth-promoting strategies in modern poultry production systems (Soren et al., 2024; Youssef et al., 2024).

## Conclusions

The present study demonstrates that dietary supplementation with a synbiotic combining SC and MOS significantly enhances growth performance, feed efficiency, intestinal morphology, hematological indices, and immune responses in broiler chickens, without adversely affecting mortality rates. Birds receiving the synbiotic diet exhibited the greatest improvements in body weight gain, feed conversion ratio, villus height-to-crypt depth ratio, and both humoral and innate immune parameters compared to those in probiotic, prebiotic, or control groups. These physiological benefits resulted in substantial economic gains, as synbiotic supplementation yielded the highest gross returns and net profits, reflecting superior cost-effectiveness. Collectively, the combined use of SC and MOS as a synbiotic represents a sustainable, antibiotic-free nutritional strategy that effectively optimizes growth, gut health, immune competence, and profitability in modern broiler production systems.

## CRediT authorship contribution statement

**Z. Li:** Writing – review & editing. **F. Raziq:** Writing – review & editing, Writing – original draft. **M. Waqas:** Data curation. **S. Khan:** Conceptualization. **T.A. Asseri:** Writing – review & editing, Writing – review & editing. **M. Mobashar:** Resources. **A. Ullah:** Funding acquisition. **H.A. Bachaya:** Writing – review & editing. **M.T. Khan:** Writing – review & editing. **N. Baazaoui:** Writing – review & editing. **M. Al-Rasheed:** Writing – review & editing. **S. Yang:** Writing – review & editing.

## Disclosures

The authors declare no conflicts of interest regarding the publication of this research article.
